# Resolution in bullous pemphigoid

**DOI:** 10.1007/s00281-019-00759-y

**Published:** 2019-11-15

**Authors:** Christian D. Sadik, Enno Schmidt

**Affiliations:** 1 Department of Dermatology, Allergy, and Venerology, University of Lübeck, Lübeck, Germany; 2 Lübeck Institute of Experimental Dermatology (LIED), University of Lübeck, Ratzeburger Allee 160, 23538 Lübeck, Germany

**Keywords:** Autoimmunity, Bullous pemphigoid, Pathophysiology, IL-17, Complement, Blistering, Resolution

## Abstract

Pemphigoid diseases are a group of autoimmune blistering skin diseases defined by an immune response against certain components of the dermal-epidermal adhesion complex. They are prototypical, autoantibody-driven, organ-specific diseases with the emergence of inflammatory skin lesions dependent on the recruitment of immune cells, particularly granulocytes, into the skin. During an acute flare of disease, inflammatory skin lesions typically progressing from erythema through urticarial plaques to subepidermal blisters erosions erupt and, finally, completely resolve, thus illustrating that resolution of inflammation is continuously executed in pemphigoid disease patients and can be directly monitored on the skin. Despite these superb conditions for examining resolution in pemphigoid diseases as paradigm diseases for antibody-induced tissue inflammation, the mechanisms of resolution in pemphigoid are underinvestigated and still largely elusive. In the last decade, mouse models for pemphigoid diseases were developed, which have been instrumental to identify several key pathways for the initiation of inflammation in these diseases. More recently, also protective pathways, specifically IL-10 and C5aR2 signalling on the molecular level and T_regs_ on the cellular level, counteracting skin inflammation have been highlighted and may contribute to the continuous execution of resolution in pemphigoid diseases. The upstream orchestrators of this process are currently under investigation. Pemphigoid disease patients, particularly bullous pemphigoid patients, who are predominantly above 75 years of age, often succumb to the side effects of the immunosuppressive therapeutics nowadays still required to suppress the disease. Pemphigoid disease patients may therefore represent a group of patients benefiting most substantially from the introduction of non-immunosuppressive, proresolving therapeutics into the treatment regimens for their disease.

## Definition of pemphigoid diseases

Organ-specific, IgG- and/or IgA-mediated immune responses are one of the most common pathomechanisms in the pathogenesis of autoimmune diseases. In humans, there are examples for this aberrant type of immune response leading to disease in almost any organ [1]. This also includes the skin where an IgG- and/or IgA-mediated immune response directed to specific components of the dermal-epidermal adhesion complex is the defining, common pathomechanistic principle of pemphigoid diseases, a group of autoimmune blistering skin diseases, which characteristically features the formation of subepidermal, dense blisters of the skin.

Seven different disease entities, namely bullous pemphigoid (BP), pemphigoid gestationis, mucous membrane pemphigoid, linear IgA disease, lichen planus pemphigoides, anti-p200 pemphigoid, and epidermolysis bullosa acquisita (EBA), belong to the group of pemphigoid disease entities. Although the diseases share many aspects of their histopathological and clinical presentation, they also partially differ in many aspects, including in their autoantigens (Table 1) and in the long-term course of disease (reviewed in [2–4]).

**Table 1:** Pemphigoid diseases and their respective associated autoantibodies

Pemphigoid disease	Autoantigens
Bullous pemphigoid (BP)	BP180 NC16A domain (type XVII collagen) BP230
Pemphigoid gestationis	BP180 NC16A domain (type XVII collagen) BP230
Lichen planus pemphigoides	BP180 NC16A domain (type XVII collagen) BP230
Epidermolysis bullosa acquisita (EBA)	Type VII collagen
Anti-p200 pemphigoid	p200 protein/laminin γ1
Mucous membrane pemphigoid (MMP)	BP180, laminin 332, BP230^a^, α6β4 integrin, type VII collagen
Linear IgA disease	LAD-1, BP230^a^

^a^In nearly all cases, autoantibodies against one of the other target antigens is found

The by far most common and best examined pemphigoid disease is BP. We will therefore focus our brief overview on the clinical features and pathophysiology pemphigoid disease on BP. More detailed information on other pemphigoid diseases are available in numerous excellent reviews published on the clinical features and management of pemphigoid diseases in the last years [1–4].

## Bullous pemphigoid

BP is the most frequent autoimmune blistering skin disease with a prevalence of approximately 21,000 patients, i.e. 260/million, in Germany [5] and an incidence varying between populations from 10 to 25 patients/million/year in Central Europe and the USA [6–10]. Registry data from the UK and Sweden even reveal incidences of about 70/million/year [11, 12]. BP is, notably, a disease of the elderly with most patients affected in the 8th and 9th decennium with a mean age at the time of diagnosis between 75 and 80 years [5, 10, 12]. Accordingly, incidences steeply rise with age to more than 200/million/year in individuals older than 80 years [6–8, 11].

BP is a chronic disease usually exhibiting an undulating, remitting-relapsing course. However, in the pre-corticosteroid era, about 70% of patients already succumbed to the first flare of disease [13]. Since the introduction of various immunosuppressants into the treatment of pemphigoid diseases, acute flares of disease can be usually therapeutically suppressed but disease relapses in 40% of patients within 6 months after the discontinuation of immunosuppressive therapy [14] and the 1-year mortality of BP patients after the first flare is approximately 20–40% and, thus, 2–3-fold that of age- and sex-matched peers [4, 14, 15]. The cause for the increased mortality of BP patients even under treatment is not entirely clear. Many patients, however, succumb to infections, which are certainly promoted by the immunosuppressive drugs employed for the treatment of BP. It appears evident that with the vast majority of BP patients being elderly above 80 years, the collective of BP patients is exceptionally susceptible to the unwanted side effects of immunosuppressive drugs.

BP is defined by the formation of autoantibodies against the hemidesmosomal protein BP180, also termed type XVII collagen (Col17) [2–4, 16]. About half of BP patients, additionally, form autoantibodies against BP230, another hemidesmosomal protein, probably due to epitope spreading, but the pathogenic significance of anti-BP230 autoantibodies is still not fully elucidated [17–19]. Like in all pemphigoid diseases, the deposition of autoantibodies at the dermal-epidermal junction (DEJ) alone does not precipitate inflammatory skin lesions. The latter require the marked recruitment of immune cells, particularly of granulocytes, into the dermis. Apart from old age, the greatest risk factors appear to be debilitating neurological disorders that affect a third to half of the BP patients and usually precede the autoimmune skin disease [20–23], and the use of diuretics, psycholeptics, and dipeptidyl-peptidase IV inhibitors (gliptins) [20, 24–28].

**Fig. 1:**
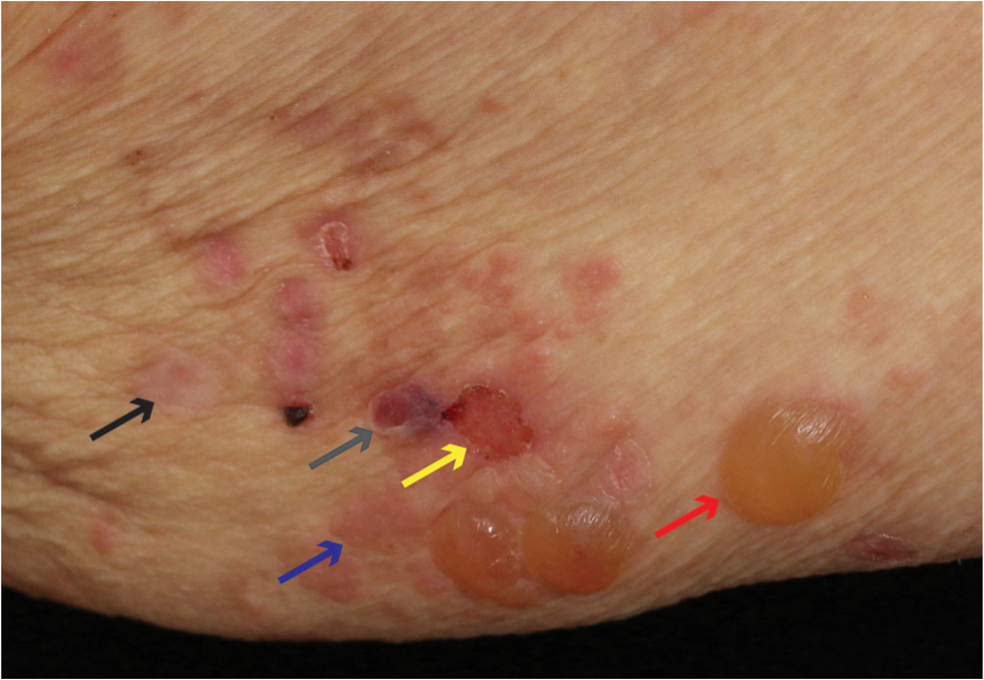
Typical co-existence of inflammatory skin lesions in different stages of development, including an emerging skin lesions in the shape of an urticarial plaque (blue arrow), a skin blister (red arrow), an erosion (yellow arrow), a resolving/healing erosion (grey arrow), and a resolved/healed skin lesions (black arrow) on the medial aspect of the upper arm of a BP patient during an acute flare

The clinical presentation of BP is variable but in its typical and most common clinical presentation, it exhibits the eruption of inflammatory skin lesions with the individual skin lesion progressing from erythema through urticarial plaques, blisters, and erosions to uninflamed and scarlessly re-epithelizing skin [2, 29] (Fig. 1). Thus, herein, the latter stage represents a state of resolving or resolved skin inflammation on track to reach the *reconstitutio ad integrum*. The different stages of lesion progression often co-exist in the individual patient during an acute flare of disease. This description of the inflammatory skin lesions in BP already implicates that, although autoantibodies usually deposit at the dermal-epidermal junction throughout the entire skin, in most cases, skin lesions do not involve the entire skin at the same time but only erupt in certain areas scattered all over the body but often sparing the face. The reasons for this selective eruption of skin lesions are still elusive. Of interest, data from the pre-corticosteroid area indicate that one third of BP patients will recover from the disease within 5 years without specific treatment [13]. Collectively, these clinical observations, however, strongly advocate the existence of endogenous, proresolving, and anti-inflammatory mechanisms first counteracting the emergence of skin inflammation and later, terminating (“resolving”) it, where it finally could erupt.

Diagnosis is based on (i) a compatible clinical picture, (ii) linear deposits of IgG and/or complement C3 at the dermal-epidermal junction by direct immunofluorescence (IF) microscopy of a perilesional skin biopsy, and (iii) the detection of serum autoantibodies against BP180 and/or BP230 [15, 30–36].

Treatment of moderate and severe BP relies on the long-term use of systemic corticosteroids usually combined with potentially corticosteroid-sparing immunomodulants such as doxycycline and dapsone or immunosuppressants such as methotrexate, azathioprine, and mycophenolate [15, 36–39]. In severe or recalcitrant patients, adjuvant rituximab, immunoadsorption, or high-dose intravenous immunoglobulins can be employed [15, 37, 40–43].

While several mouse models of pemphigoid diseases have been instrumental in uncovering some of the key proinflammatory pathways driving the effector phase of these diseases, research into these protective mechanisms is still in its infancy. The few insights gained to this point into these protective mechanisms will be summarized following a brief update on the pathophysiology of BP.

## Pathophysiology

Pathology in BP is driven by autoantibodies against the two hemidesmosomal proteins BP180 (also termed type XVII collagen, Col17) and BP230 (reviewed in [2–4, 16, 44]). In addition, to anti-Col17 IgG about 40 and 60% of BP patients develop IgE and IgA anti-Col17 reactivity, respectively [18, 45–51]. BP230 is recognized by 50–70% of BP sera [32, 34, 52–56].

While in BP, a large body of evidence has been assembled to describe the pathogenic importance of autoantibodies and various mechanisms that mediate tissue destruction of anti-Col17 IgG (detailed below), data about the cellular immune response, undoubtedly essential in every autoimmune disease, are rather scare [57]. T and B cell reactivity against the NH_2_-terminal portion of the BP180 ectodomain is associated with severe BP, while the central portion is more frequently recognized in patients with limited disease. In contrast, combined T and B cell response against the COOH- and NH_2_-terminal globular domains of BP230 were found in less than 50% [58]. The response to the Col17 ectodomain is restricted to the DQβ1*0301 allele [59, 60]. Autoreactive T cells in BP patients release a Th1/Th2 mixed cytokine profile [58, 59]. While the number of circulating CD4+CD25+FoxP3+ regulatory T cells, natural killer T cells, and natural killer cells are normal, γδ T cell numbers were reported to be reduced in BP patients [61, 62].

A plethora of data has been published about the pathogenic relevance of anti-Col17 antibodies, while only conflicting reports were available about the pathogenicity of anti-BP230 IgG. Recently, however, the injection of monoclonal BP230-specific IgG in neonatal mice induced macroscopic and microscopic blistering suggesting their pathogenic potential [63, 64].

Our knowledge about the events that lead to subepidermal blistering upon binding of anti-Col17 antibodies to their cell surface receptor on keratinocytes is derived from the observation that serum levels of Col17NC16A-specific IgG antibodies correlate with the disease activity in BP patients [65–70] as well as various experimental models. Latter models include the incubation of cultured human keratinocytes with Col17-specific IgG/IgE, the treatment of cryosections of human skin with Col17-specific IgG followed by incubation with normal human leukocytes, and, importantly, various mouse models of BP and BP-like inflammatory EBA [71–77] (reviewed in [78–81]).

**Fig. 2:**
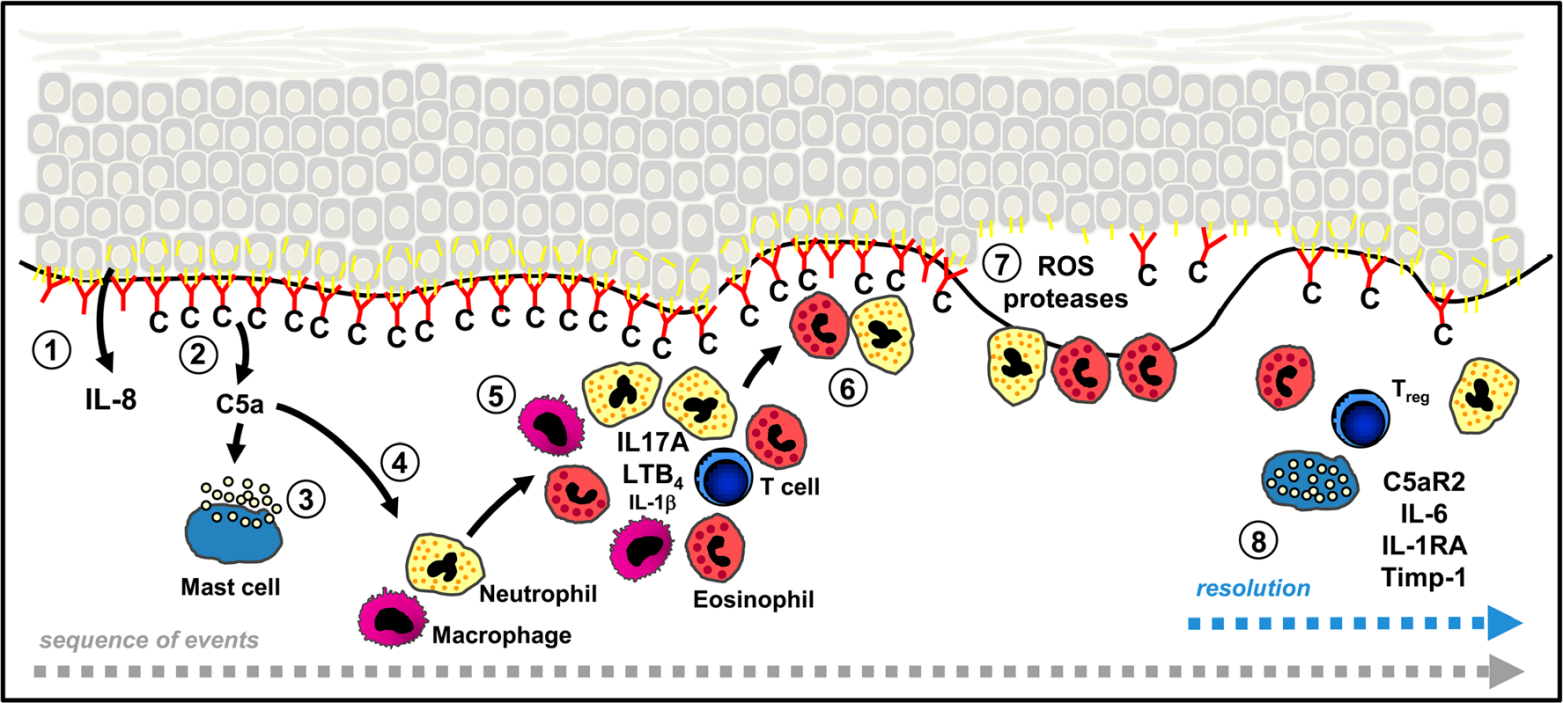
Sequence of events leading to dermal-epidermal separation and potentially proresolving pathways in bullous pemphigoid (BP). Binding of autoantibodies (red) against type XVII collagen (Col17, yellow) initiates Fc-independent events, e.g. the release of IL-8 from basal keratinocytes (1). Complement is activated at the dermal-epidermal junction (DEJ) and C5a released (2). Mast cells degranulate after binding of C5A or anti-Col17 IgE (3) and inflammatory cells attracted by C5a appear in the upper dermis (4, 5). Their secretion of additional inflammatory mediators such as IL-17A, LTB4, and to a lesser extent, of IL-1β further increases and maintains the inflammatory reaction. Subsequently, neutrophils and eosinophils line along the DEJ (6) and release reactive oxygen species and specific proteases that ultimately induce dermal-epidermal separation (7). In animal models of BP and BP-like epidermolysis bullosa acquisita, the anti-inflammatory effect of regulatory T cells (T_reg_), C5aR2, and IL-6 (via IL-1 receptor antagonist, IL-1RA, and tissue inhibitor of metalloproteinase-1, Timp-1) was shown and point to potentially proresolving mechanisms in BP. Modified from [4] and [3]

Based on these models, the following sequence of events has appeared (Fig. 2). While most effects depend on the Fc-portion of autoantibodies, also, very early in the diseases course, Fc-independent effects have been described including the release of IL-6 and IL-8 from keratinocytes following binding of anti-Col17 IgG or IgE [72, 82] as well as internalization of Col17 and weakening of keratinocyte attachment in response to anti-Col 17 IgG [83–87].

The earliest Fc-dependent effect of autoantibodies is most likely the activation of complement at the dermal-epidermal junction, an event observed in the skin of nearly all BP patients. Complement activation leads to the influx of inflammatory cells such as neutrophils, eosinophils, and macrophages [75, 88–90]. Complement-derived anaphylatoxins such as C5a have strong chemotactic effects on these cells. Here, both the classical and the alternative pathway of complement activation are important and most effects are most likely exerted via the C5aR1 on leucocytes [88, 89, 91]. In addition, the degranulation of mast cells, one of the earliest events observed in BP lesions, may be exerted via C5aR1 on mast cells [92]. An even more striking usage of C5aR1 was observed in mouse models of BP-like inflammatory EBA and anti-laminin 332 mucous membrane pemphigoid [93–96].

In addition to mast cells [90, 92], neutrophils [97–101], eosinophils [102], and macrophages [90] were shown to contribute to subsequent tissue destruction. So far, only few individual inflammatory mediators with a striking effect on the autoantibody-mediated tissue destruction have been identified in addition to C5a, including LTB_4_ and IL-17A.

Leukotriene B_4_ (LTB_4_) is another chemoattractant critically involved in the regulation of neutrophil influx into the skin. Thus, deficiency in 5-lipoxygenase, the rate-limiting enzyme in the biosynthesis of LTB_4_, or in the LTB_4_ receptor BLT1 confers dramatic resistance to the recruitment of neutrophils into the skin and, consequently, to the emergence of skin lesions in both the antibody transfer BP-like EBA and BP mouse model [103, 104]. Furthermore, scavenging of LTB_4_ by the drugs Coversin and PAS-L-Coversin significantly attenuates disease [105]. In line with this critical role of LTB_4_ in the first stages of lesion development, LTB_4_ levels in the start increasing briefly after the injection of anti-Col7 antibodies into the skin [103]. Tissue resident cells, such as macrophages, are presumably the initial cellular sources of LTB_4_. This notion, however, still requires clarification. Once recruited into the skin, neutrophils themselves are an abundant source of LTB_4_ and amplify their recruitment into the skin in a manner similar to what was previously demonstrated for autoantibody-induced inflammation in other organs [104, 106–108].

CD4-positive cells were identified as the major source of IL-17A both in the blood and early skin lesions of BP patients compared to age- and sex-matched controls. mRNA analysis of early skin lesions revealed *IL17A* and related cytokines and chemokines to be significantly upregulated. IL17A-deficient mice were greatly protected by the otherwise pathogenic effect of anti-Col17 IgG compared to wild-type animals, and anti-Col17 IgG-injected mice developed significantly fewer clinical lesions when treated with an anti-IL17 A antibody compared to isotype control antibody-treated mice [109]. In addition, Antonicelli and coworkers showed that (i) IL-17 serum levels are lower in patients in remission compared to the time when treatment was initiated, (ii) IL-17A is involved in the formation of neutrophil extracellular traps in the BP skin lesions, and (iii) IL-17 induce the release of neutrophil elastase and matrix metalloproteinase-9 from normal human polymorphonuclear cells [110–112].

C5a and LTB_4_ may thus induce the influx of inflammatory cells in the upper dermis, while IL-17 may orchestrate the inflammatory reaction in the skin that finally leads to blister formation.

In early BP lesions, neutrophils and eosinophils are found to line up along the dermal-epidermal junction. Reactive oxygen species and specific proteases such as matrix metalloproteinase-9 and neutrophil elastase were shown to be released form infiltrating leucocytes and lead to dermal-epidermal splitting [71, 113, 114]. Although the proteolytic activity most likely not specifically targets individual basement membrane proteins, matrix metalloproteinase-9 and neutrophil elastase were found in blister fluid and lesional biopsies of BP patients and were capable to degrade Col17 [115–117]. In fact, the importance of individual proteases was quite well studied in the neonatal mouse model of BP [98–101, 118, 119]. It appears that in the early stages of blistering, matrix metalloproteinase-9 is mainly activated by plasmin, which is formed by activation of plasminogen by tissue plasminogen activator and/or urokinase plasminogen activator. Plasmin and the mast cell-specific serine protease-4 can activate matrix metalloproteinase-9 which then inactivates α1-proteinase inhibitor, the physiological inhibitor of neutrophil elastase. The unrestrained activity of neutrophil elastase is then responsible for the degradation of structural proteins of the dermal-epidermal junction including Col17 [98–101, 118, 119]. This cascade of events is further amplified and perpetuated by the activation of the coagulation cascade by eosinophils, which further promotes the recruitment of eosinophils into the dermis [44, 120, 121].

In summary, some aspects of BP physiology, such as the sequence of events leading to blistering, including the requirement of autoantibodies and the infiltration of inflammatory cells, have been relatively well defined. Further studies will focus on the trigger factors that induce the generation of anti-Col17 and anti-BP230 antibodies in BP and on the identification of pharmacological inhibitors of key inflammatory mediators and pathways.

## Resolution

Some mediators have been described that are present in the blood and/or skin of BP patients and were shown to exert anti-inflammatory properties when their functional relevance was explored in mouse models of BP or BP-like EBA. Below we summarize the current knowledge about the so far identified anti-inflammatory factors in BP including C5aR2, IL-6, and IL-10, and discuss them as effector molecules in the resolution of proresolving potential.

### Complement activation

Both, the alternative and, to even a larger extent, the classical pathway were shown to be important for blister formation. In the neonatal mouse model of BP, activation of the classical pathway was even reported to be a requisite for dermal-epidermal separation in this model. Mice deficient in C5 or C1q did not develop blisters upon injection of anti-Col17 IgG due to the lack of neutrophil recruitment to the skin [88, 89, 122]. The quasi dogma of complement dependency of BP was later questioned when the passive transfer of F(ab’)_2_ fragments of human Col17-specific IgG or anti-Col17 IgG4 led to skin fragility when injected in Col17-humanized mice. In the same model, the induction of skin fragility upon injection of Col17-specific IgG in C3-deficient mice pointed in the same direction [122–124]. In the antibody transfer model of BP employing adult mice, the injection of anti-Col17 IgG in C5-deficient mice halved the extent of skin lesions as compared to wild-type mice independent of the IgG dose [91]. In line, when C5aR1-deficient mice were injected with anti-Col17 IgG, significantly less skin lesions arose compared to wild-type animals; however, C5aR1-deficient mice still developed about two third of the extent of lesions induced in wild-type mice [91]. These results indicate that although complement activation is an important factor to recruit leucocytes to the upper dermis, BP lesions may develop independently of complement activation.

Interestingly, when Col17-specific IgG was injected in C5aR2-deficient mice, these mice developed significantly more skin lesions compared to wild-type animals. While C5aR1 clearly is a proinflammatory mediator, the role of C5aR2 in inflammation appears to be multifaceted and may depend on the individual disease and the stage of inflammation [125]. In various disorders, such as sepsis, immune-complex-mediated lung injury, and allergic contact dermatitis, like in BP, C5aR2 has a protective role [125]. In line, migration of bone marrow-derived C5aR1-deficient neutrophils towards recombinant C5a was significantly lower compared to neutrophils from C57BL/6 wild-type mice, while migration of neutrophils from C5aR2-deficient animals was similar to neutrophils from wild-type mice [91]. Current research within the Clinical Research Unit 303 *Pemphigoid Diseases* aims at further delineating the anti-inflammatory and potentially proresolving role of C5aR2 in BP.

### Interleukin-6

IL-6 is a pleiotropic cytokine that has been identified as key proinflammatory mediator in several diseases including rheumatoid arthritis, Castleman’s disease, Takayasu arteritis, and giant cell arteritis [126]. However, proinflammatory effects of IL-6 have been described, e.g. in animal models of endotoxic lung disease and endotoxemia [127]. The classical signalling pathway is initiated by binding of IL-6 to IL-6R and a second transmembrane protein, gp130, which serves as a signal transducer of IL-6. IL-6 can also bind to soluble IL-6Rs (sIL-6Rs forming an IL-6-sIL-6R complex that can bind to membrane-bound gp130 on cells that do not express IL-6R on the surface, a process known as trans-signalling) [126, 128]. In a mouse model of BP-like EBA, we have shown that blockade of IL-6 led to significantly increased skin lesions via classical IL-6 signalling most likely by the inhibition of IL-1R antagonist [129]. More specifically, while patients as well as anti-Col7 IgG-injected mice revealed elevated levels of IL-6 in blister fluids (BP patients only), sera and skin, IL-6-deficient mice or mice treated with an blocking anti-IL-6 antibody developed significantly higher disease activity compared to wild-type animals [129, 130]. In contrast, treatment with recombinant IL-6 dose-dependently impaired the induction of experimental BP-like EBA and led to increased expression of IL-1R antagonist in skin and serum [129]. Co-injection of mice with anti-Col7 IgG and the IL-1R antagonist anakinra significantly reduced the induction of skin lesions [129]. Latter data were later corroborated by the finding that after disease induction by the immunization of susceptible SJL/J mice with recombinant Col7, anakinra prevented disease progression compared to phosphate buffer saline (PBS)-treated mice [131]. In line, IL-1R-deficient or IL-1β-deficient mice were significantly less susceptible to the skin lesion-inducing effect of anti-Col7 IgG [131]. In addition to interfering with IL-1 homeostasis, IL-6 may exert its anti-inflammatory role in experimental BP-like EBA by the observed increased dermal expression of tissue inhibitor of metalloproteinase-1 (Timp-1; a physiological inhibitor of metalloproteinase) in IL-6-treated mice [129]. Metalloproteinase-9 has previously been shown to be essential for blister formation in the neonatal mouse model of BP (see above) [100].

### T_regs_ and Interleukin-10

On the cellular level, T_regs_ have been highlighted to promote the timely resolution of skin lesions in the antibody transfer BP-like EBA mouse model. Thus, depletion of T_regs_ in DEREG significantly aggravated and prolonged disease [132]. Supporting also a similar role of T_regs_ in the human situation, T_regs_ are also present in lesional skin of BP patients. Notably, their density in lesional BP skin is, however, significant lower than in psoriasis and atopic dermatitis suggesting a relative deficiency in T_regs_ in BP [133], which may contribute to the persistence of skin inflammation in pemphigoid diseases. The mechanisms T_regs_ apply to counteract skin inflammation are still largely elusive, but there is first evidence for a significant role of IL-10 in this process. Thus, the induction of IL-10^+^ plasma cells efficiently suppressed skin inflammation in the antibody transfer BP-like EBA mouse model, among other, by inducing the release of IL-10 from T_regs_ in the skin [134]. Intriguingly, a series of in vitro experiments suggested that IL-10 may suppress disease in pemphigoid diseases by directly inhibiting the effect of C5a on neutrophils [134].

### Proresolving lipid mediators

Multiple lines of evidence predominantly generated in mouse models of acute peritonitis, pouchitis, ischemia, or colitis point at a central role of proresolving lipid mediators as orchestrators of resolution [135–137]. The validity of this principle for the resolution of autoantibody-induced tissue inflammation is, however, still uncertain [135], and the role of proresolving lipid mediators in pemphigoid diseases has not been investigated either. However, profiling the lipidome in emerging skin lesions in the antibody transfer mouse model of BP-like EBA, we detected 12/15-lipoxygenase-derived proresolving lipid mediators [103], suggesting that 12/15-lipoxygenase is already active in the initiation phase of skin lesions and might counteract their emergence from the very beginning. Whether the enzyme is still active during the resolution of skin lesions is currently under investigation.

## Concluding remarks

In recent years, a number of anti-inflammatory pathways counteracting skin inflammation in pemphigoid diseases have been uncovered. These pathways may also play a role in the resolution of skin inflammation in pemphigoid diseases which still requires detailed investigation. With single skin lesions in BP continuously emerging and completely resolving, BP appears as excellent model to dissect the mechanisms of resolution in autoantibody-induced tissue inflammation and injury. In that line, the disease may potentially also respond particularly well to proresolving therapeutic strategies, and, with pemphigoid disease patients often succumbing to the side effects of immunosuppressive drugs, these diseases may be among those benefiting the most from the introduction of proresolving therapies into treatment regimens.
